# Kinetic Modeling of Slightly Acidic Electrolyzed Water Decay Characteristics in Fresh Cabbage Disinfection Against Human Norovirus

**DOI:** 10.3389/fmicb.2021.616297

**Published:** 2021-07-06

**Authors:** Miran Kang, Boyeon Park, Ji-Hyoung Ha

**Affiliations:** Hygienic Safety and Analysis Center, World Institute of Kimchi, Gwangju, South Korea

**Keywords:** cabbage, disinfection, kinetic model, norovirus, SAEW decay

## Abstract

To consistently disinfect fresh vegetables efficiently, the decay of disinfectants such as chlorine, electrolyzed oxidizing water (EOW), ozonated water, and plasma-activated water during the disinfection maintenance stage needs to be understood. The aim of our study was to evaluate the changes in the inactivation kinetics of slightly acidic electrolyzed water (SAEW) against human norovirus (HuNoV), based on the cabbage-to-SAEW ratio. After disinfection of fresh cabbage with disinfected SAEW solution, SAEW samples were collected and analyzed for physicochemical properties such as pH, available chlorine concentrations (ACCs), and oxidation-reduction potential (ORP). SAEW virucidal effects were evaluated. We confirmed the decay of post-disinfection SAEW solution and demonstrated the different patterns of the decay kinetic model for HuNoV GI.6 and GII.4. In addition, the goodness of fit of the tested models based on a lower Akaike information criterion, root-mean-square error (RMSE), and residual sum of squares (RSS) was close to zero. In particular, the change in both the HuNoV GI.6 and GII.4 inactivation exhibited a strong correlation with the changes in the ACC of post-disinfection SAEW. These findings demonstrate that physicochemical parameters of SAEW play a key role in influencing the kinetic behavior of changes in the disinfection efficiency of SAEW during the disinfection process. Therefore, to optimize the efficiency of SAEW, it is necessary to optimize the produce-to-SAEW ratio in future studies.

## Introduction

The food industry has recognized the importance of hygiene practices and has established optimized processes to ensure food safety. For cleaning and sterilization processes, chemical compounds such as organic disinfectants like ethanol, chlorine compounds, iodine, quaternary ammonium compounds, and hydrogen peroxide have been utilized in various food processes (Hricova et al., 2008). Among chemical disinfection processes, chlorination is the most efficient; however, it can lead to the generation of potentially toxic halogenated disinfection by-products (DBPs) and corrode metal (Issa-Zacharia et al., 2011). The use of some chlorination treatments has been restricted due to discoloration or unpleasant odor of food products. Therefore, alternative disinfectants such as electrolyzed oxidizing water (EOW), ozonated water, and plasma-activated water have been evaluated for food safety and quality (Schwarz et al., 2019).

Slightly acidic electrolyzed water (SAEW) can be generated by electrolysis of sodium chloride or hydrochloric acid using a non-membrane electrolytic cell (Li et al., 2013). Recently, SAEW treatment with low available chlorine concentrations (ACCs), usually approximately 30 mg/L (the acceptable range is between 10 and 80 mg/L), and a pH value between 5.0 and 6.0, has been gaining attention as a disinfectant in the food industry to remove pathogenic bacterial populations on food-contact surfaces or food products (Suzuki et al., 2005). In various studies, the antibacterial effects of SAEW on foodborne pathogenic bacteria, such as *Staphylococcus aureus*, *Salmonella enteritidis*, and *Escherichia coli*, have been evaluated (Fenner et al., 2006; Ren and Su, 2006; Hao et al., 2014; Cap et al., 2019). The virucidal effects of electrolyzed water (EW) were investigated in human norovirus (HuNoV) GII.4 Sydney and cultivable HuNoV surrogates, for example, and murine norovirus in suspension and on stainless steel surfaces has also been evaluated (Park et al., 2007; Moorman et al., 2017). Moreover, SAEW and sodium hypochlorite treatment have an equivalent disinfection efficacy in fresh-cut cilantro, spinach, and cut cabbage samples (Koide et al., 2009; Rahman et al., 2010; Hao et al., 2011). Furthermore, Rahman et al. (2013) and Wang et al. (2014) demonstrated that SAEW treatment can be utilized to disinfect fresh shrimp and pork. Based on these experimental results, the United States Environmental Protection Agency (EPA) has approved the use of EW generators for disinfection in the food processing field. Moreover, the Japanese Ministry of Health, Labor and Welfare has also authorized EW as a food additive to reduce pathogenic microbial populations in various foods, food contact surfaces, and food processing surfaces.

Between pH 5.0 and 6.5, the most common active form of chlorine compounds among hypochlorite ions (ClO^–^), chlorine gas (Cl_2_), and hypochlorous acid (HOCl) is HOCl (95%), which induces pathogenic microbial inactivation. The relative amounts of the HOCl, Cl_2_, and ClO^–^ species formed in SAEW solutions is pH dependent; therefore, changes in pH have a significant effect on the formation of chlorine compounds. Previous studies demonstrated that the SAEW is most effective in eliminating pathogenic microorganisms at a pH of approximately 5.5, when the proportion of HOCl is the highest (Hricova et al., 2008). Moreover, a specific oxidation-reduction potential (ORP), which indicates the ability to oxidize or reduce, has been reported to be the main factor influencing the antimicrobial activity of SAEW (Al-Haq et al., 2005). Thus, the antimicrobial effect of SAEW is influenced by specific factors such as pH, ACC, and ORP (Fabrizio and Cutter, 2004).

Maintaining SAEW treatment efficacy should be considered in disinfection processes for fruits or vegetables from the perspective of the relationship between disinfection parameters. The changes in the physicochemical properties of SAEW play an important role in ensuring the antimicrobial activity during the disinfection process. In disinfectant processes, previous studies pay more attention to the inactivation efficacy for pathogens; however, the decay of SAEW in the disinfection maintenance stage was not considered important. Recently, with the increase of the use of SAEW in washing and sanitization of fruits and vegetables, the decay behavior of SAEW in solution is gradually gaining attention. Therefore, understanding the properties of SAEW decay as cleaning agent and disinfectant could help to optimize SAEW disinfection treatment and help maintain disinfection efficiency in the food industry.

The objectives of our study were to investigate the changes in virucidal effects based on the operating variables of SAEW treatment in fresh cabbage. In this study, we investigated how much the deterioration of post-disinfection SAEW affects its ability to disinfect against HuNoV. Moreover, we evaluated kinetic models to predict the changes in the physicochemical properties of SAEW and the deterioration of virucidal effects on HuNoVs. Finally, we determined the effect of cabbage-to-SAEW ratio as an operating variable on the disinfection of fresh cabbage by post-disinfection SAEW.

## Materials and Methods

### Virus Stocks

HuNoV genogroup I genotype 6 (HuNoV GI.6) and genogroup II genotype 4 (HuNoV GII.4) were provided by Norogene (Seoul, South Korea). The HuNoV GI.6 and GII.4 stock samples were diluted in nuclease-free water (Ambion Inc., Austin, TX, United States) and vortexed briefly. The solution was stored in 500-μl aliquots at −80°C.

### Preparation and Characterization of SAEW

The SAEW was prepared from electrolysis of 7% hydrochloric acid, using a SAEW generator (Purester m-Clean; Morinaga Milk Industry Co., Ltd., Tokyo, Japan) at 12.8 V and 5.0 A. A dual-scale pH/mV meter (Accumet AB15; Fisher Scientific, Fair Lawn, NJ, United States) equipped with bearing ORP and pH electrodes was used to determine the ORP and pH values of the prepared solution. The ACC was measured using a colorimetric method of a digital chlorine test kit (RC-3F; Kasahara Chemical Co., Saitama, Japan). The SAEW generation rates were achieved at a flow rate of 10.0 L/min. Initial SAEW had an ORP of 975 ± 2 mV, a pH range of 5.46 ± 0.01, and an ACC of 26.65 ± 0.84 ppm. After production, the SAEW was placed in a stainless-steel tank developed in the lab for disinfection. The laboratory scale of SAEW tank is 1.6 m (length), 0.8 m (depth), and 0.8 m (width).

### Virucidal Effect of the Post-disinfection SAEW

**FIGURE 1:**
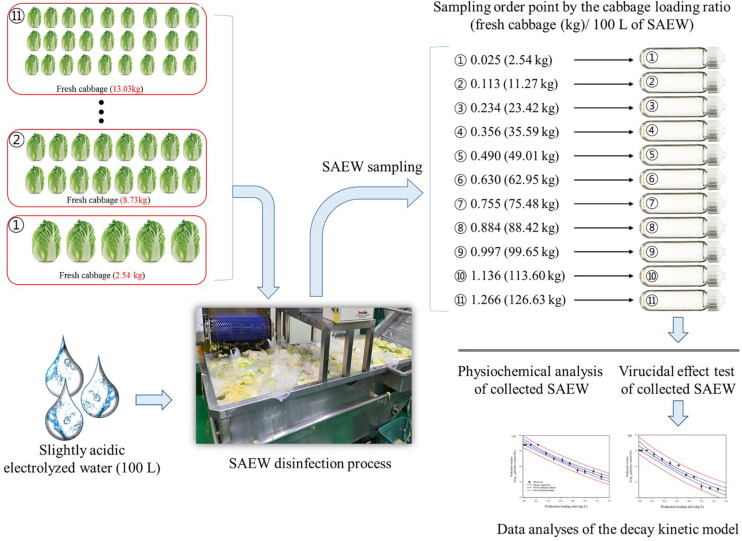
Schematic diagram of the SAEW sampling for virucidal test of HuNoV.

A schematic diagram of SAEW sampling for the HuNoV virucidal test in the experimental study is shown in Figure 1. For testing the virucidal effects of post-disinfection SAEW, 100 L of SAEW (fixed volume) and 50 samples of fresh cabbage (*Brassica rapa* L. subsp. *pekinensis*) were prepared. The cabbages were purchased from a supermarket in Gwangju, South Korea, and pre-sorted at 5°C and bruised outer leaves were removed and discarded. Each cabbage sample was weighed and recorded to calculate the ratio of cabbage (weight) to SAEW (volume). SAEW was collected for the virucidal test immediately after the weighed cabbage was sequentially washed in SAEW. Samples of post-disinfection SAEW solutions were collected and denoted by the ratio [fresh cabbage (kg)/100 L of SAEW] of disinfecting trial at 0.025 (2.54 kg), 0.113 (11.27 kg), 0.234 (23.42 kg), 0.356 (35.59 kg), 0.490 (49.01 kg), 0.630 (62.95 kg), 0.755 (75.48 kg), 0.884 (88.42 kg), 0.997 (99.65 kg), 1.136 (113.60 kg), and 1.266 (126.63 kg) disinfecting order, and assayed for virucidal effect. All experiments were performed twice. A modified Dilution-Neutralization Method (European CEN EN 1,276 method) was used to evaluate the viral disinfection efficacy of post-disinfection SAEW. A quantitative suspension test of viral disinfection was performed as previously described by Ha et al. (2016). Briefly, 0.1 ml of stock suspension of HuNoV (containing approximately 1.0E + 5 to 2.2E + 5 genomic RNA copies) was added into 0.9 ml of each post-disinfection SAEW solution, based on the designated ratio. Phosphate buffered saline (PBS, pH 7.4, Sigma-Aldrich) was used as a negative control, and 2% sodium hypochlorite adjusted to pH 7.0 (Sigma Aldrich) was used as a positive control. Then, they were quickly vortexed and incubated for 60 s at 20 ± 2°C, as suggested in the CEN EN 1276 protocol. Immediately following the SAEW contact time, 10 ml of PBS was added to the mixture to neutralize the disinfection reaction.

### Quantification of HuNoV in Suspension Test

HuNoV GI.6 and GII.4 concentration, elution, and quantification from SAEW disinfected sample groups were conducted as previously described by Lee et al. (2018). The following optimum method for quantification of HuNoV RNA particles from SAEW-disinfected samples was used: an anionic polymer-coated magnetic bead separation (MBS)/reverse transcription quantitative polymerase chain reaction (RT-qPCR) method with pretreatment combining sodium lauroyl sarcosinate (INCI) and propidium monoazide (PMA) (denoted as MBS/INCI/PMA/RT-qPCR assay). Lee et al. (2018) used 200 μM PMA to quantify HuNoV GI.6 and GII.4 viral RNA. The optimum concentration of 0.5% INCI, which caused minimal damage to intact HuNoVs viral particles, was used. HuNoV RNA was precipitated by mixing the viral solution with 97% ethanol and mixed with AVL extraction buffer and carrier RNA. A QIAamp spin column (Qiagen, Hilden, Germany) was used to purify the viral RNA sample. After two wash steps with AW1 and AW2 buffers, 60 μl of elution buffer (AVE) was used to elute the viral RNA. A 5-μl aliquot of each test sample was placed in a PCR tube directly for RT-qPCR. HuNoV viral particles were recovered using commercial Viro-adembeads (Ademtech, Pessac, France) after disinfection tests. The beads were prepared by coating with poly(methyl vinyl ether-maleic anhydride), and virus capture was conducted according to the manufacturer’s procedure. A 5 mg/ml (final concentration) of nanosized magnetic bead mixture was added to the disinfected HuNoV viral suspension and shaken for 1 h at 20 ± 2°C to combine the viral particles. The bead mixture with combined HuNoV viral particles was recovered using the PolyATtract System 1000 (Promega, Madison, WI, United States) and resuspended in 100 μl of PBS.

### Viral RNA Extraction and RT-qPCR

Viral RNA was eluted with 60 μl of AVE buffer using a QIAamp MinElute virus spin kit (Qiagen, Hilden, Germany) and then purified RNA was used immediately to avoid template RNA freezing. A 5-μl aliquot of each RNA test sample was used directly in a one-step RT-qPCR (7500 Fast Real Time PCR System, Applied Biosystems, Foster City, CA, United States) using the QuantiTect Probe RT-PCR kit (Qiagen). For HuNoV GI.6 and GII4, RT-qPCR was performed using 5 μl of RNA elute in a total volume of 20 μl with the following cycling parameters: 50°C for 600 s, denaturation at 95°C for 300 s, and 40 cycles of amplification with denaturation at 95°C for 10 s and combined annealing and extension at 60°C for 30 s. NoV GI primer (10 pmol each) sequences were 5′ -JJV1R TCCTTAGACGCCATCATCAT-3′ (reverse) and JJV1F 5′ -GCCATGTTCCGITGGATG-3′ (forward), which were used to amplify the 96-base pair (bp) fragment of the NoV GI polymerase gene. The TaqMan probe (JJV1P) sequence was FAM 5′ -TGTGGACAGGAGATCGCAATCTC-3′ BHQ (Gentry et al., 2009). The NoV GII.4 primer sequences (10 mM each) were COG2R: 5′-TCGACGCCATCTTCATTCACA-3′ and COG2F: 5′-CARGARBCNATGTTYAGRTGGATG AG-3′, which amplified a 122-bp fragment of the NoV GII.4 (Lee et al., 2018). The TaqMan probe (Ring2, 10 mM) was FAM: ′-TGGGAGGGCGATCGCAATCT-3′ BHQ. All amplifications were performed in triplicate.

### Quantification of NoV RNA

To plot external standard curves of HuNoV GI.6 and GII.4, quantitative synthetic norovirus G1 (I) RNA (ATCC^®^ VR3234SD^TM^, ATCC, Manassas, VA, United States) and G2 (II) RNA (ATCC^®^ VR3235SD^TM^) containing the ORF1- and ORF2-junction region was sequentially diluted 10-fold from 2.0 to 6.0 log_10_ genomic RNA copy numbers. The standard RNA curves of both NoV GI.6 and GII.4 had slopes of −3.264 (*R*^2^ = 0.9953) and −3.371 (*R*^2^ = 0.9981), respectively. The initial titers of NoV GI.6 and GII.4 were approximately 5.31 and 5.08 log_10_ genomic copies per RT-qPCR reaction, respectively.

### Data Analyses of the Decay Kinetic Model

Reduction values of HuNoV viral titer in log10 was calculated as cabbage washing accumulated over all trials, and two mathematical decay kinetic models were fitted to the average HuNoV viral titer using the SigmaPlot software (version 14.0, San Jose, CA, United States) with the decay kinetic models (Eq. 1). This equation is expressed below:

(1) Reduction⁢values⁢of⁢HuNoV=a×exp⁢(b×(cabbage⁢loading⁢ratio)),

where *a* and *b* are regression parameters. For the sum of squares optimization, the Akaike information criterion (AIC) formula (Eq. 2) was used as expressed below:

(2) $$A\,I\,C=n\times l\,n\,\left(\frac{s\,s}{n}\right)\times 2\times k$$

where *n* is the number of observations, *k* is the number of estimated parameters in the model, and *ss* is the sum of squares. The root-mean-square error (RMSE) of the mathematical kinetic model prediction with respect to the estimated variable *Y*_model_ is defined as the square root of the mean squared error (Eq. 3):

(3) $$\text{RMSE}=\sqrt{\frac{\sum \frac{n}{i=1}\,{\left(Y_{o\,b\,s,i}-Y_{m\,o\,d\,e\,l,i}\right)}^{2}}{n-k}}$$

where *n* is the number of observations, *k* is the number of estimated parameters in the model, *Y*_obs_ is the observed value, and *Y*_model_ is the modeled value at cabbage loading ratio *i*. The residual sum of squares (RSS) is the sum of the squared distances between predicted values versus actual treated data:

(4) $$\text{RSS}=\sum_{i=1}^{n}{\left(Y_{o\,b\,s,i}-Y_{m\,o\,d\,e\,l,i}\right)}^{2}$$

where *Y*_obs_ is the observed value and *Y*_model_ is the modeled value at cabbage loading ratio *i*.

### Statistical Analysis

Duplicate samples were used in each operating variable (cabbage-to-SAEW ratio), and the experiments were repeated in triplicate. For statistical analysis, one-way ANOVA and Duncan’s multiple range test were used to compare differences among mean values using SPSS Statistics (software v.8.2, Inc., Chicago, IL, United States). *P*-value < 0.05 was defined as significant. The experimental results are denoted as log_10_ genomic copies/μl, and regression analysis was performed using the SigmaPlot software system (ver. 14.0).

## Results

### Changes in the Physicochemical Properties of SAEW

**FIGURE 2:**
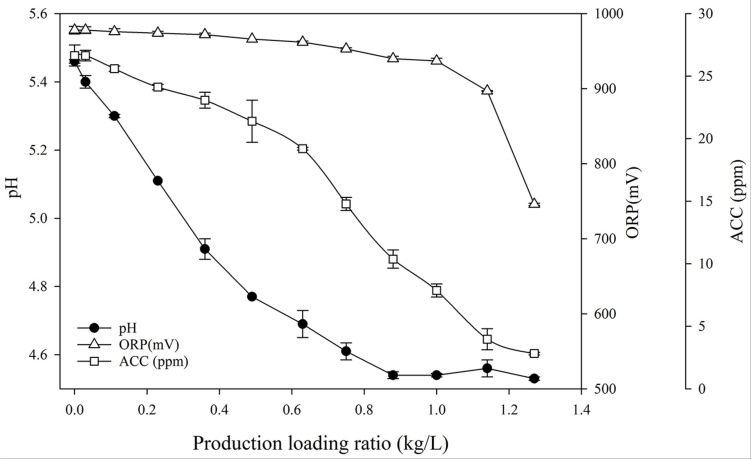
Changes in the pH, ORP, and ACC of SAEW sampled after disinfection and washing fresh cabbages.

We investigated whether the ratio of fresh cabbage loaded to the fixed volume of SAEW affected the physicochemical characteristics of SAEW. After disinfection and washing, the SAEW solution was sampled according to the specified product loading ratio (kilogram of fresh cabbage per liter of SAEW; kg/L). The changes in pH, ORP, and ACC of SAEW sampled after disinfection and washing fresh cabbages are shown in Figure 2. As the product loading ratio increased, the pH value decreased significantly. When the cabbage/SAEW ratio was 0.3, the pH was 5.0. Interestingly, when the cabbage/SAEW ratio was more than 0.3, the pH of SAEW began to deviate from the effective disinfection range (between 5.0 and 6.0). This was a section where the reduction of the disinfection effect could be estimated. In addition, when the cabbage/SAEW ratio was 0.3, ORP maintained a level of approximately 980 mV without significant differences, and ACC maintained a level of 23.6 ppm. ACC values had reduced by 3 ppm from the initial value of 26.6 ppm. Thereafter, the pH value and ACC exhibited a tendency to decrease continuously. When the cabbage/SAEW ratio was 0.87, the decrease in the pH value stopped at 4.54. On the other hand, the ORP maintained a level of 940 mV without significant differences until the cabbage/SAEW ratio was 1.0, and then rapidly changed to less than 900 mV as the cabbage/SAEW ratio exceeded 1.0.

### Decay Kinetic Model of Virucidal Effects of Post-disinfection SAEW on HuNoV

**FIGURE 3:**
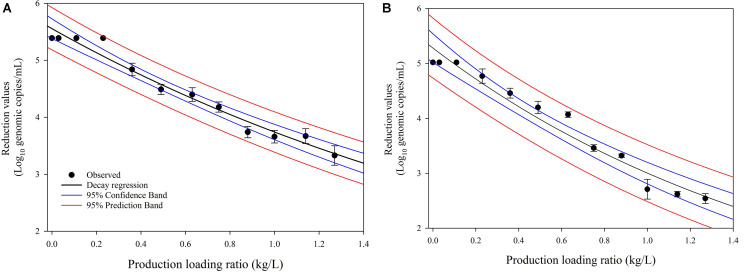
Dynamic changes of the virucidal efficacies of **(A)** HuNoV GI.6 and **(B)** HuNoV GII.4 based on the cabbage loading ratio related to the total amount of SAEW.

We investigated the dynamic changes in the virucidal efficacies of SAEW affected by cabbage disinfection and washing operating variables (cabbage-to-SAEW ratio). The cabbage loading ratio significantly affected the dynamic changes in the virucidal efficacies on HuNoV GI.6 and HuNoV GII.4 (Figures 3A,B). Increasing the amount of fresh cabbage washed in the same washing chamber with 100 L of SAEW resulted in a significant decrease in the reduction values of both HuNoV strains. Moreover, it led to a decrease in the pH value of the SAEW solution. The most interesting and novel findings in the present study are shown in Figures 3A,B. Although the initial disinfection and washing process showed excellent elimination of both HuNoV GI.6 and GII.4, with a reduction of approximately 5.02–5.32 log genomic copies, the obtained reduction values significantly and continuously diminished as the cabbage loading ratio increased. In particular, the reduction value of HuNoV GII.4 decreased to less than 3 log decrease as the cabbage/SAEW ratio exceeded 1.0, whereas the reduction value of HuNoV GI.6 maintained a 3 log decrease. Based on these data, we assumed that HuNoV GII.4 might be more resistant than HuNoV GI.6 to SAEW treatment. These data indicate that SAEW treatment loses efficiency as the cabbage concentration increased.

### Model Evaluation With Experimental Data

The three model selection criteria used to investigate the correspondence between the obtained kinetic models are presented in Table 1. Generally, values close to zero for AIC, RMSE, and RSS indicate a better goodness-of-fit model. Thus, the lowest value obtained from the model selection criterion implies the preferred kinetic model. The decay kinetic model of virucidal effects on HuNoV by post-disinfection SAEW shows that the goodness-of-fit of the tested models are based on lower AIC values in both HuNoV strains. The RMSE values in the decay kinetics were 0.0217 (HuNoV GI.6) and 0.0452 (HuNoV GII.4). In addition, the RSS values from decay kinetics appeared to be appropriate for inactivation of both HuNoV strains.

**TABLE 1:** Analysis of SAEW decay kinetic models in terms of reduction values of HuNoVs based on cabbage-to-SAEW ratio.

**Fitting model (decay kinetics)**		**Parameter statistical distributions**			
		**^1)^*R*^2^**	**^2)^AIC**	**^3)^RMSE**	**^4)^RSS**
	
HuNoV GI.6	*Y* = 5.5565 × exp(−0.3952 × *X*)	1.017	1.017	0.0217	0.2166
HuNoV GII.4	*Y* = 5.2817 × exp(−0.5646 × *X*)	2.377	2.377	0.0465	0.4652

*^(1)^*R*^2^ is the coefficient of determination.*

*^(2)^AIC is the Akaike information criterion.*

*^(3)^RMSE is the root-mean-square error.*

*^(4)^RSS is residual sum of squares.*

### Pearson Correlation Coefficient Between Virucidal Effect and Physicochemical Parameters

The Pearson correlation coefficients between the reduction value of HuNoV GI.6 and pH, ORP, and ACC were 0.945, 0.680, and 0.966, respectively, and those between the reduction of HuNoV GII.4 and the above three factors were 0.835, 0.789, and 0.991, respectively (Table 2). Overall, pH and ACC were strongly correlated with a reduction in both HuNoV strain titers (*P* < 0.05). ACC showed the strongest correlation with the inactivation of HuNoV GI.6 (*R* = 0.966) and HuNoV GII.4 (*R* = 0.991).

**TABLE 2:** Correlation coefficients between reduction value of HuNoVs and the values of pH, ORP, and ACC.

**Reduction values (log_10_ genomic copies)**	**Physicochemical properties**		
	**pH**	**ORP (mV)**	**ACC (ppm)**
	
HuNoV GI.6	0.945	0.680	0.966
HuNoV GII.4	0.835	0.789	0.991

## Discussion

### Effect of MBS-PMA-INCI Pretreatment on RT-qPCR Assay

Reverse transcription quantitative polymerase chain reaction has been used extensively to quantify and detect the viral particles owing to its sensitivity, specificity, and short assay time. Nevertheless, there is technical limit to the use of nucleic acid-based detection techniques for the assessment of viral particles after disinfection: their inability to distinguish infectious and non-infectious viruses in disinfected samples. MBS can be utilized to overcome this issue. The MBS technique for improving the sensitivity of virus concentration and detection has been verified in numerous studies (Tian et al., 2008; Morton et al., 2009; Pan et al., 2012). Moreover, Moore et al. (2016) reported that nucleic acid aptamers, as valuable ligands, can help discern the infectivity status of viral particles. In addition, there have been many new studies on differentiating intact viral particles using more advanced analysis techniques such as PMA or ethidium monoazide (EMA) pretreatments (Coudray-Meunier et al., 2013; Escudero-Abarca et al., 2014; Moreno et al., 2015; Randazzo et al., 2016). The intercalating dye eliminates false-positive results caused by the nonspecific binding to magnetic beads (Sánchez et al., 2012; Sangsanont et al., 2014). Therefore, the MBS/INCI/PMA/RT-qPCR assay used in this study is a reliable and suitable technique to capture and discrimination intact, damaged, and dead viral particles after SAEW disinfection.

### Changes in the Physicochemical Properties of SAEW

For optimal disinfection, SAEW solution should be checked continuously, before and after the disinfection process, to prevent decrease of concentration below the disinfection limit of SAEW. However, there has been little research on the dynamics of the changes in physicochemical properties of SAEW during vegetable disinfection, although various studies have shown changes in the physicochemical properties (e.g., pH, ORP, and ACC) of EW in suspension (Tatsumi et al., 2007; Cui et al., 2009). Suboptimal disinfection conditions of EW due to changes in the pH, ORP, and ACC could reduce the microorganism inactivation (Park et al., 2004; Al-Haq et al., 2005). Len et al. (2002) reported that the major factor influencing the antimicrobial activity of acidic EW (AEW) is the fraction of chlorine compounds, Cl_2_ and HOCl, and subsequent HOCl decomposition. Furthermore, bactericidal activities of chlorine compounds are pH dependent (Fabrizio and Cutter, 2004; Mahmoud et al., 2004). As a type of EW, the virucidal effect of SAEW also reacts sensitively to changes in pH, ORP, and ACC. In our study, we found a strong correlation between virucidal activity and physicochemical parameters. These experimental data suggest that physicochemical factors of SAEW play a crucial role in the kinetic behavior of changes in SAEW during the disinfection process. ACC plays a more important role than pH and ORP in reducing foodborne pathogens (Hotta et al., 1994; Koseki et al., 2004; Kim et al., 2006). Hsu and Kao (2004) demonstrated that the primary reason for deterioration of EOW antimicrobial activity may be decomposition of chlorine compounds rather than changes in pH or ORP. Moreover, ACC in EOWs can be easily converted to the inactive form by changes in the EOW environment (Tamaki et al., 2014). Our findings also showed the strongest correlation coefficient between deterioration in virucidal activity of SAEW and the pattern of ACC decay.

### Decay of Virucidal Effects of Post-disinfection SAEW on HuNoV

Several studies have investigated decay kinetic models of chlorine for public water distribution systems (Hua et al., 1999; Le Dantec et al., 2002) and activated sludge (Caravelli et al., 2003). Li et al. (2013) reported chlorine decay kinetic models for AEW during storage against foodborne pathogens such as *Vibrio parahaemolyticus* and *Listeria monocytogenes* in suspension. However, to date, there have been few studies regarding the decay kinetics of the virucidal effects of SAEW during disinfection and wash treatment of fresh cabbage. Furthermore, most studies on the kinetics of inactivation based on chlorine conditions were dependent on exposure time (Le Dantec et al., 2002; Caravelli et al., 2003). However, the decay of SAEW during the disinfection maintenance stage was not sufficient during the actual disinfection process. In the present study, we determined the virucidal decay behavior based on the change in the ratio of cabbage load to the total SAEW volume. In particular, this study was performed at a pilot scale to evaluate the real washing and disinfection processes. Our results indicated that increasing the amount of fresh cabbage for washing in fixed 100 L of SAEW resulted in a significant decrease in the reduction of both HuNoV GI.6 and GII.4. This demonstrates the continuous accumulation of organic substances from washed cabbage in the SAEW, leading to SAEW deterioration. Our results are consistent with those of Tamaki et al. (2014). The effectiveness of the various types of EOWs could be considered as short term because highly reactive oxidative moieties in EOWs would react with any organic compound present during the cabbage washing process. Furthermore, ACC in EOWs can be quickly altered to the inactive form. Therefore, our results regarding decay kinetic models of SAEW for washing and disinfection systems provide experimental data to effectively sterilize and wash cabbage using SAEW. The developed exponential decay model for HuNoVs reduction by treating sampled SAEW after the washing process of fresh cabbage gave statistically acceptable fits. In the kinetic models, the model selection criteria, including RSS, AIC, and RMSE based on goodness of fit, indicated an acceptable goodness of fit of both the HuNoV GI.6 and HuNoV GII.4.

The organic load from wash water is a crucial parameter that affects the inactivation activities of disinfectant washing treatment. Like other oxidative disinfectants, fractions of chlorine compounds are highly reactive to organic materials and could be rapidly deteriorated by organic matter in the disinfection agent (Gil et al., 2009). In particular, Luo et al. (2012) demonstrated that a significant number of organic substances, including exudate from surfaces of vegetable debris, soil, and various material particles, are transferred into the disinfectant solution. This causes rapid decay of disinfectant quality, manifested by changes in pH, ORP, and ACC. Luo et al. (2012) reported that the washing process achieved lower bacterial reduction with larger produce-to-wash water ratio. According to Pirovani et al. (2001), the produce-to-water ratio that would allow efficient microbial inactivation is not clear, and the microbial reduction achieved depends on the operating conditions (e.g., water-to-produce ratio, disinfectant condition, exposure time, and temperature) and the type of vegetables. Therefore, several studies have evaluated operational variables such as water-to-produce ratio and washing time. Pirovani et al. (2001, 2004) reported that a higher produce-to-water ratio can significantly reduce microbial inactivation. Similarly, in our study, the reduction of both HuNoVs was significantly and continuously diminished as the cabbage loading ratio increased. Our results indicated that inactivation efficacy of post-disinfection SAEW on both HuNoVs strains was affected by the cabbage-to-SAEW ratio. Furthermore, the deterioration of inactivation levels in post-disinfection SAEW used in this study was similar to what was demonstrated in the decay pattern of post-disinfectant water samples obtained from a fresh-cut lettuce processing plant (Nou et al., 2011).

### Virucidal Effects of EW

Although it is known that pH, chlorine compounds, ORP, or combinations of these variables have a significant role in the antimicrobial activity of SAEW, there is no evidence that they are associated with inactivation of foodborne or waterborne viruses. Hati et al. (2012) reported that the antimicrobial mechanisms of AEW could be primarily attributed to three variables: availability of chlorine compounds, ORP, and pH. The virucidal effect of AEW has been evaluated for herpes simplex viruses, hepatitis B virus, human immunodeficiency virus, and enteric viruses (Tanaka et al., 1999; Morita et al., 2000; Tagawa et al., 2000; Park et al., 2007). Moreover, Moorman et al. (2017) reported that neutral electrolyzed water (NEW) at 250 ppm had virucidal effects on HuNoV GII.4 in suspension and on stainless steel surfaces. Furthermore, NEW showed promising results as a virucidal treatment when applied on food contact surfaces. Tamaki et al. (2014) reported that both H5N1 and H9N2 viruses were inactivated by 5 log 60 s after the viruses were treated with AEW, in which the ACC ranged from 0 to 72 ppm. Interestingly, Tamaki et al. (2014) demonstrated that AEW influences viral inactivation effectiveness in an ACC-independent manner and for a relatively long period. However, in our study, it is noteworthy that in both HuNoVs strains, inactivation efficacy of post-disinfection SAEW is ACC dependent. The AEW produced in the anode chamber of an EW generator generally presents high ORP and low pH values, unlike SAEW. Interestingly, it was demonstrated that the pH value of AEW was inversely proportional to the ORP value (Al-Haq et al., 2002), and decreasing the pH increased the virucidal potential of AEW, even if chlorine compounds (HOCl, Cl_2_, and ClO^–^) were kept constant (Park et al., 2004). However, the pH value of SAEW was proportional to the ACC and ORP values, respectively, in our results. This was unexpected because both EW and SAEW are substances with similar chemical properties. This discrepancy may be attributed to the organic substances from the washed cabbages. Furthermore, differences in various food matrices among fresh vegetables could lead to different results for the virucidal effects of SAEW.

In this study, our experimental data demonstrated that physicochemical parameters of SAEW play a key role in describing the kinetics of changes in SAEW properties during the cabbage disinfection process. The three selection criteria, AIC, RMSE, and RSS, used to determine the correspondence between the obtained kinetic models were close to zero, indicating statistically acceptable fits. Furthermore, our findings showed a strong correlation coefficient with the deterioration in the virucidal effect of SAEW and the changes in pH, ACC, and ORP. Therefore, for SAEW to be used efficiently in the pre-treatment for washing and disinfection of fresh vegetables, it is necessary to optimize the produce-to-SAEW ratio as operating variables in future studies.

## Data Availability Statement

The original contributions presented in the study are included in the article/supplementary material, further inquiries can be directed to the corresponding author.

## Author Contributions

MK and BP performed most of the experiments, analyzed the data, and wrote the first draft of the manuscript. J-HH was responsible for the experimental design, data coordination, analysis and interpretation, and writing, revision, and finalization of the manuscript. All authors have read and approved the final manuscript.

## Conflict of Interest

The authors declare that the research was conducted in the absence of any commercial or financial relationships that could be construed as a potential conflict of interest.
